# Cofilin Oligomer Formation Occurs *In Vivo* and Is Regulated by Cofilin Phosphorylation

**DOI:** 10.1371/journal.pone.0071769

**Published:** 2013-08-08

**Authors:** Pankaj Goyal, Dharmendra Pandey, Daniela Brünnert, Elke Hammer, Marek Zygmunt, Wolfgang Siess

**Affiliations:** 1 Institut für Prophylaxe und Epidemiologie der Kreislaufkrankheiten, Klinikum Innenstadt, Universität München, München, Germany; 2 Klinik und Poliklinik für Frauenheilkunde und Geburtshilfe, Universitätsmedizin, Greifswald, Greifswald, Germany; 3 Interfakultäres Institut für Genetik und Funktionelle Genomforschung, Universitätsmedizin Greifswald, Greifswald, Germany; Astar-Neuroscience Research Partnership (NRP) and Institute of Medical Biology (IMB), Singapore

## Abstract

**Background:**

ADF/cofilin proteins are key regulators of actin dynamics. Their function is inhibited by LIMK-mediated phosphorylation at Ser-3. Previous *in vitro* studies have shown that dependent on its concentration, cofilin either depolymerizes F-actin (at low cofilin concentrations) or promotes actin polymerization (at high cofilin concentrations).

**Methodology/Principal Findings:**

We found that after *in vivo* cross-linking with different probes, a cofilin oligomer (65 kDa) could be detected in platelets and endothelial cells. The cofilin oligomer did not contain actin. Notably, ADF that only depolymerizes F-actin was present mainly in monomeric form. Furthermore, we found that formation of the cofilin oligomer is regulated by Ser-3 cofilin phosphorylation. Cofilin but not phosphorylated cofilin was present in the endogenous cofilin oligomer. *In vitro*, formation of cofilin oligomers was drastically reduced after phosphorylation by LIMK2. In endothelial cells, LIMK-mediated cofilin phosphorylation after thrombin-stimulation of EGFP- or DsRed2-tagged cofilin transfected cells reduced cofilin aggregate formation, whereas inhibition of cofilin phosphorylation after Rho-kinase inhibitor (Y27632) treatment of endothelial cells promoted formation of cofilin aggregates. In platelets, cofilin dephosphorylation after thrombin-stimulation and Y27632 treatment led to an increased formation of the cofilin oligomer.

**Conclusion/Significance:**

Based on our results, we propose that an equilibrium exists between the monomeric and oligomeric forms of cofilin in intact cells that is regulated by cofilin phosphorylation. Cofilin phosphorylation at Ser-3 may induce conformational changes on the protein-protein interacting surface of the cofilin oligomer, thereby preventing and/or disrupting cofilin oligomer formation. Cofilin oligomerization might explain the dual action of cofilin on actin dynamics *in vivo*.

## Introduction

Actin dynamics regulate cell functions. For example, actin cytoskeleton rearrangement is essential for platelet shape change, secretion, and aggregation, and it underlies endothelial cell contraction, migration, and proliferation [1]–[4]. Actin polymerization and depolymerization are highly regulated processes. Various signaling events such as phosphorylation, Ca^2+^, and pH modulate the properties of a variety of actin binding proteins (ABPs). The actin depolymerization factor ADF/cofilin family of proteins (15–19 kDa) is expressed in all eukaryotic cells and is essential for enhancing actin filament turnover. These proteins weakly sever actin filaments without capping their ends, thereby increasing the number of free filament ends where polymerization and depolymerization occur. They also enhance the rate of monomer dissociation from the pointed ends [5]. ADF/cofilin proteins not only promote the disassembly of ADP-actin monomers from filaments, but they also bind to release ADP-actin monomers and inhibit the exchange of their bound nucleotides [6].

Besides actin filament depolymerization, cofilin can also accelerate spontaneous assembly of actin monomers [7], and activation of microinjected caged cofilin increases actin polymerization in carcinoma cells [8]. Notably, enhancement of actin polymerization activities has not been reported for ADF.

The function of cofilin is regulated by LIM-kinase mediated phosphorylation at Ser-3 [9]. Phosphorylation of cofilin inhibits its binding to actin [10]. We, as well as others, have found that in cells stimulated by the same stimulus, cofilin phosphorylation is regulated differentially: Stimulation of endothelial cells with thrombin increased cofilin phosphorylation [11]. In contrast, stimulation of platelets with thrombin led to a rapid dephosphorylation of cofilin. Cofilin dephosphorylation in platelets induced by either thrombin stimulation or inhibition of the Rho-kinase/LIMK1 pathway was associated with an increase of F-actin [12], [13].

A previous study showed that cofilin possesses an intrinsic tendency to form oligomers *in vitro* mediated by disulphide bonds, and that in contrast to the monomer, cofilin oligomers are unable to depolymerize actin filaments but rather induce actin polymerization and filament bundling [14]. In line with these studies, an *in vitro* analysis has shown that the function of cofilin depends on the concentration of cofilin: low cofilin concentrations favor actin filament severing, whereas high cofilin concentrations favor actin polymerization [7].

In the present study, we addressed the question of whether cofilin oligomer formation occurs *in vivo* and, if so, whether it is regulated by cell stimulation. We also pondered the possible role of LIMK-mediated cofilin phosphorylation and phosphatase-mediated cofilin dephosphorylation in the regulation of cofilin oligomerization. Our results indicate that cofilin exists as both a monomer and an oligomer in endothelial cells and platelets. Rho-kinase/LIMK-mediated phosphorylation of cofilin at Ser-3 inhibited cofilin oligomerization *in vitro* and *in vivo,* whereas cofilin dephosphorylation enhanced cofilin oligomerization *in vivo*.

## Results

### Cofilin exists as a monomer and an oligomer in endothelial cells and platelets

**Figure 1:**
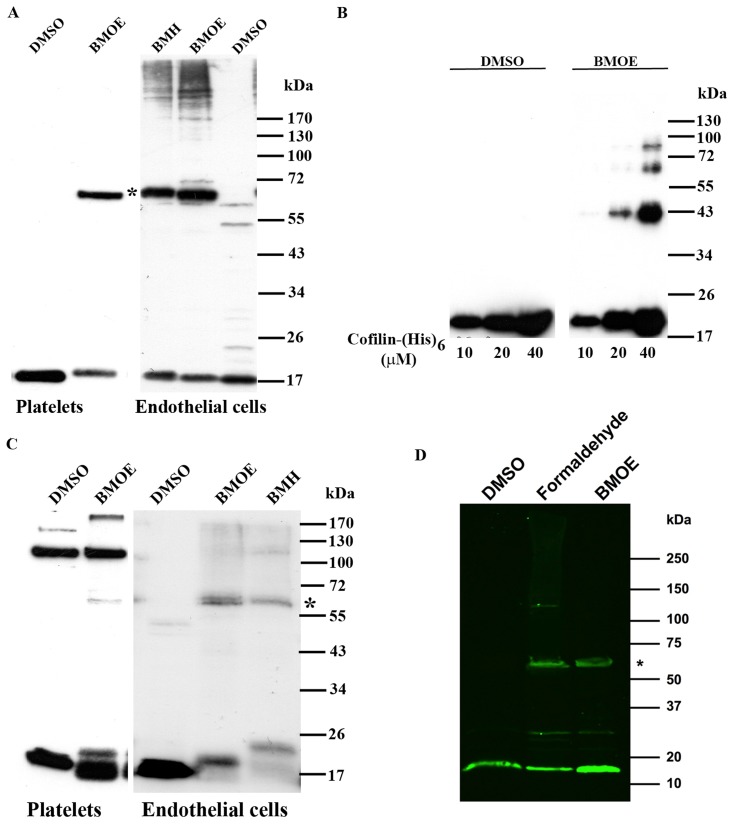
Cofilin exists as an oligomer *in vivo* and *in vitro*. A) Human washed platelets (4×10^8^/ml, 0.4 ml) and endothelial cells (0.8–1×10^6 ^cells/20 µl) were incubated with DMSO (1 µl) or membrane-permeable, non-cleavable, homobifunctional, maleimide cross-linkers (BMOE and BMH) at a final concentration of 1 mM and 0.2 mM, respectively. The cell lysates were immunoblotted with anti-cofilin antibody. A cofilin oligomer (∼65 kDa; indicated by asterisk) was observed in BMOE and BMH but not in DMSO-treated platelets and endothelial cells. B) Recombinant His-cofilin at concentrations ranging from 10 µM to 40 µM was incubated with BMOE (two fold molar excess of cofilin) or treated with DMSO for one hour at room temperature. BMOE- and DMSO-treated cofilins were subjected to SDS-PAGE and analyzed by coomassie blue staining. C) The cross-linked platelet and endothelial cells lysates (as described in A) were immunoblotted with anti-ADF antibody. A faint band (∼65 kDa) was observed in lysates of BMOE and BMH but not in DMSO-treated cells. D) Endothelial cells (0.8–1×10^6 ^cells/20 µl) were incubated with DMSO (1 µl) or BMOE (1 mM). For formaldehyde cross-linking, endothelial cells (1×10^6 ^cells/ml) were treated with formaldehyde at a final concentration of 1%. The cell lysates were subjected to SDS-PAGE on a gradient gel (4–15%) and were then immunoblotted with anti-cofilin antibody. A cofilin oligomer (∼65 kDa; indicated by asterisk) was observed in BMOE- and formaldehyde-treated cells, but not in DMSO-treated endothelial cells. Proteins were detected by fluorescence imaging of secondary antibodies labeled with infrared dyes.

To explore whether cofilin forms oligomers *in vivo*, we used membrane-permeable homobifunctional, maleimide cross-linkers of different lengths (BMOE and BMH) for conjugation between sulfhydryl groups (-SH). BMOE and BMH probes have spacer arms of 8Åand 13Å in length, respectively. Human cofilin has four cysteine residues at positions 39, 80, 139, and 147. By using these probes, we optimized the *in vivo* cross-linking experiments by varying the concentration of cross-linkers and the incubation times. We found that cofilin exists as a monomer and an oligomer in endothelial cells and platelets (Figure 1A). Based on the molecular mass of the cross-linked complex (∼65 kDa), the oligomer could be a cofilin tetramer. To confirm the existence of cofilin oligomers *in vivo*, we used formaldehyde, which is a different homobifunctional cross-linker: it is amine-based and acts in a reversible manner. It is membrane permeable, and has a very short spacer arm in the range of 2.3–2.7Å. We performed formaldehyde and BMOE cross-linking of endothelial cells in parallel and compared the results (see figure 1D). We found that cross-linked ∼65 kDa cofilin oligomer was present in the presence of both formaldehyde and BMOE suggesting that the ∼65 kDa cofilin oligomer does not depend on the cross-linker used. Using recombinant (His)_6_-tagged cofilin, we found that cofilin formed oligomers *in vitro* after BMOE cross-linking only at high concentrations (>10 µM) of cofilin. The (His)_6_-tagged cofilin oligomers showed different molecular masses (Figure 1B). The main oligomer had a molecular mass of 43 kDa, corresponding to a cofilin dimer. At a high cofilin concentration (40 µM), two further oligomers, one of 62–65 kDa (cofilin tetramer), and a second of 80–85 kDa (cofilin pentamer were observed (Figure 1B). Therefore, in contrast to the results of *in vitro* cross-linking experiments (Figure 1B), cofilin seems to form only a tetramer in endothelial cells and platelets *in vivo*.

### ADF mainly exists as a monomer in endothelial cells and platelets

Human ADF and cofilin both show 72% sequence homology, and ADF is also similar to cofilin as it can depolymerize actin filaments [15], [16]. We found that ADF is expressed in both platelets and endothelial cells (Figure 1C). In order to see whether ADF can form oligomers in these cells, BMOE and formaldehyde were used for cross-linking *in vivo*. We observed a weak cross-linked band of molecular mass ∼62–65 kDa in endothelial cells and platelets, which displayed only weak immunostaining with anti-ADF antibody (see asterisk; Figure 1C). Additionally, in contrast to cofilin (Figure 1D), we did not find a distinct 65 kDa ADF oligomer after cross-linking of endothelial cells, only a smear of ADF cross-linked products was observed when the BMOE and formaldehyde cross-linked endothelial cell lysates were electrophoresed on a (4–15%) gradient gel (Figure S2). These data indicate that in intact cells, ADF has, if any, only a weak tendency to form an ADF tetramer; it apparently interacts with many other proteins of various molecular weights.

### Endogenous cofilin has intramolecular disulphide bonds

**Figure 2:**
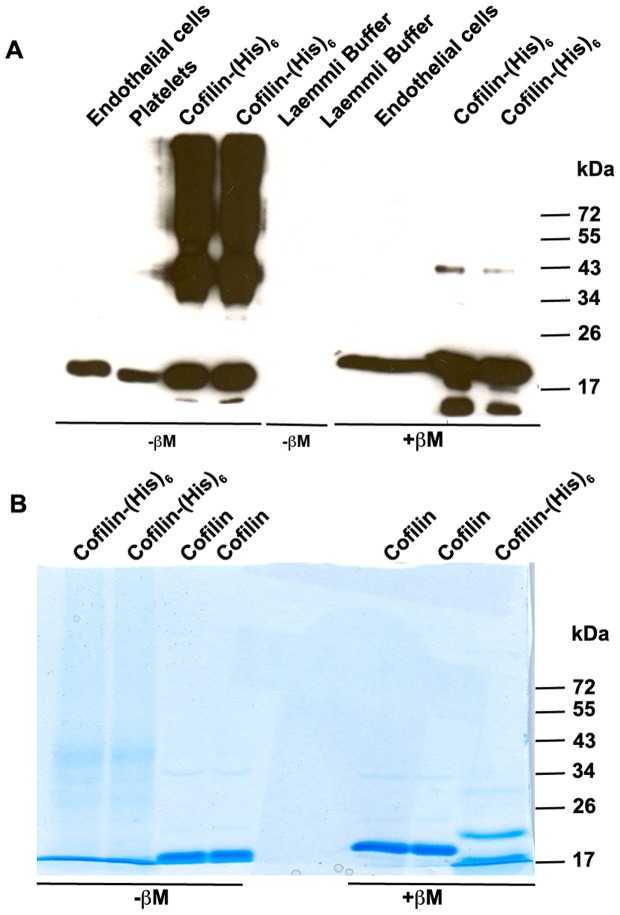
Endogenous and recombinant human His-cofilin has an intracellular disulphide bond. A) Recombinant human His-cofilin, platelet, and endothelial cell lysates were subjected to SDS-PAGE under reducing (with ß-mercapto-ethanol; +ßM) and non-reducing conditions (without ß-mercapto-ethanol; -ßM) and then immunoblotted with anti-cofilin antibody. Monomeric cofilin showed higher electrophoretic mobility under non-reducing conditions than under reducing conditions. Moreover, oligomers of different molecular masses of recombinant His-cofilin but not of endogenous cofilin were observed under non-reducing condition, suggesting the involvement of intermolecular disulphide bonding in *in vitro* oligomerization. B) Recombinant human cofilin (with and without His-tag) were subjected to SDS-PAGE under reducing and non-reducing conditions and analyzed by Coomassie blue staining. Both types of recombinant cofilin demonstrate a higher electrophoretic mobility under non-reducing conditions as compared to reducing conditions.

A previous study showed that cysteine residues of cofilin could form intramolecular disulphide bonds after oxidation [17]. In order to analyze whether or not cofilin has intramolecular disulfide bonds, platelet and endothelial cell lysates were analyzed for a mobility shift of cofilin during SDS-PAGE under reducing and non-reducing conditions and then compared with the electrophoretic mobility of recombinant (with or without His-tag) cofilin. The results showed that endogenous and recombinant cofilin exhibited higher electrophoretic mobility under non-reducing conditions than under reducing conditions (Figure 2). These data suggest that endogenous cofilin has intramolecular disulphide bonds in its normal physiological state.

Notably under non-reducing but not under reducing conditions, recombinant His-cofilin appeared as dimers and oligomers. In contrast, we could not detect endogenous cofilin oligomer in endothelial cells and platelets under non-reducing conditions, suggesting that an intermolecular disulphide bond is not needed for cofilin oligomerization in intact cells. These data indicate that intermolecular disulphide bonds are involved in cofilin oligomerization only *in vitro* but not in intact cells. This conclusion is supported by our results of the presence of ∼65 kDa cofilin oligomer in endothelial cells after treatment with formaldehyde which is an amine-based, not thiol-based cross-linker.

### The cofilin oligomer in endothelial cells and platelets does not contain actin

**Figure 3:**
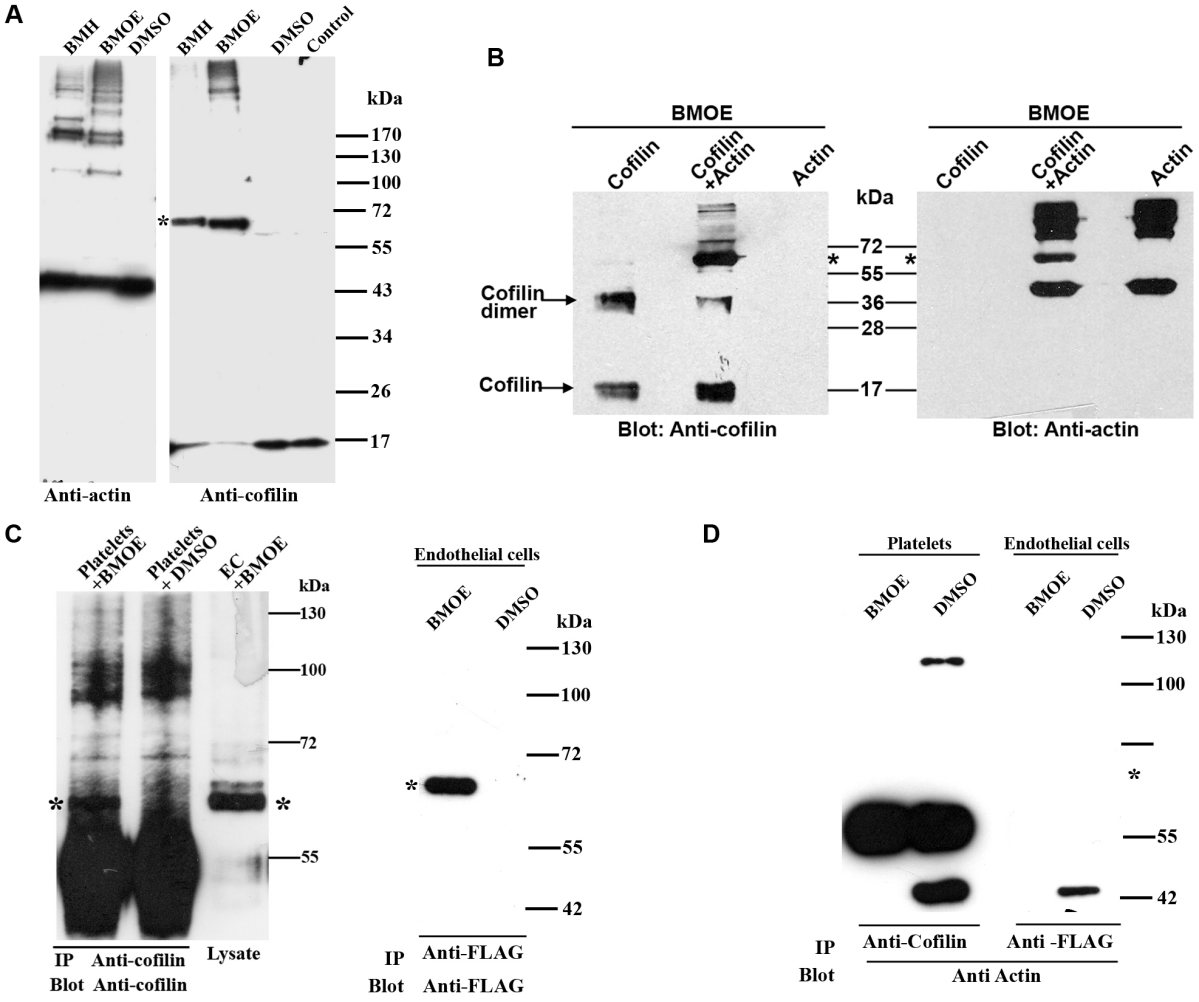
The 65 kDa cofilin oligomer in endothelial cells and platelets does not contain actin. A) Lysates of cross-linked endothelial cells (as described in Figure 1A) were immunoblotted with an anti-cofilin antibody or an anti-actin antibody. A cofilin oligomer (∼65 kDa; indicated by asterisk) was observed in BMOE and BMH but not in DMSO-treated endothelial cells (right). An actin band of 43 kDa was observed in all cell lysates. No actin could be detected at the corresponding position of the cofilin oligomer (indicated by asterisk; left). B) Equimolar concentrations (20 µM) of cofilin and actin were incubated in PBS for one hour at room temperature, then cross-linked with BMOE and immunoblotted with anti-cofilin or anti-actin antibody. Both anti-cofilin antibody (left panel) and anti-actin antibody (right panel) were able to recognize a band of cross-linked actin-cofilin heterodimer (∼62 kDa; indicated by asterisk). C) Platelets and endothelial cells transfected with FLAG-tagged cofilin were incubated with BMOE, and cross-linked cofilin oligomers were immunoprecipitated with anti-cofilin antibody (platelets) or anti-FLAG antibody (endothelial cells). The immunoprecipitated cross-linked cofilin oligomer in platelets is indicated by an asterisk (left lane; left panel), which is absent in DMSO-treated platelets (middle lane). The cross-linked oligomer in BMOE-treated endothelial cell lysate (EC) is indicated by an asterisk (right lane). The immunoprecipitated cross-linked FLAG-tagged cofilin oligomer in transfected endothelial cells samples was observed in BMOE- but not in DMSO- treated cells (right panel; indicated by asterisk). D) The immunoprecipitated samples from platelets and endothelial cells were immunoblotted with an anti-actin antibody. No actin band could be detected at the corresponding position (∼65 kDa) of the cofilin oligomers (indicated by asterisk).

We next addressed the question, whether actin might be present in the cofilin oligomer observed *in vivo* in endothelial cells and platelets. After *in vivo* cross-linking of proteins in intact endothelial cells, we could not observe actin in the cofilin oligomer by immunoblotting with a specific anti-actin antibody; instead, we found actin-cross-linked protein complexes of much higher molecular weight (Figure 3A). To test whether BMOE can cross-link actin and cofilin and whether the anti-actin antibody is able to recognize actin-cofilin cross-linked products, pure actin and cofilin proteins were incubated alone or together in the presence of BMOE. Both the anti-cofilin antibody and the anti-actin antibody detected cofilin and actin in the actin/cofilin cross-linked complex of ∼62 kDa, representing probably an actin/cofilin heterodimer (see asterisk), and in several other actin-cofilin hetero-oligomers of higher molecular weight (Figure 3B). These results indicate that the anti-actin antibody is able to recognize actin in the *in vitro* actin/cofilin cross-linked complex of 62 kDa but that actin is not present in the *in vivo* cross-linked 65 kDa cofilin oligomer.

In order to further support this conclusion, the following experiments were performed. We immunoprecipitated the cofilin oligomer from platelets and the FLAG-cofilin oligomer from FLAG-cofilin transfected endothelial cells treated with or without BMOE probe, and the immunoprecipitates were blotted after SDS-PAGE with the anti-actin antibody. Anti-cofilin and anti-FLAG antibodies were able to immunoprecipitate endogenous cofilin oligomer and FLAG-tagged cofilin oligomer from BMOE-treated platelets and endothelial cells (Figure 3C). We could not find any actin in the immunoprecipitated endogenous cofilin oligomer and FLAG-tagged cofilin oligomer, respectively (Figure 3D). These data indicate that the endogenous cofilin oligomer does not contain actin in endothelial cells.

### Identification of cofilin-interacting proteins by mass spectrometry

To identify possible cofilin-interacting proteins, cofilin was immunoprecipitated from formaldehyde cross-linked endothelial cell lysates, and the cofilin immunoprecipitates, which consisted of several cofilin-containing protein bands (including the 65 kDa cofilin oligomer) were subjected to mass spectrometry. Beside of actin we could identify 14-3-3ζ (27.7 kDa) in the cofilin immunoprecipitates. It is however, unlikely that these proteins are part of the 65 kDa cofilin oligomer: 14-3-3ζ is known to interact only with phospho-cofilin [18], which is not present in the cofilin oligomer (see below) and actin has been excluded as component of the 65 kDa cofilin oligomer (see above). Therefore, we suggest that the ∼65 kDa cofilin band is a cofilin oligomer. The detection of actin in the cofilin immunoprecipitates is not surprising, since actin was present along with cofilin in protein complexes of high Mw (>170 kDa; see actin and cofilin immunoblots in Fig. 3A).

### Cofilin phosphorylation at Ser-3 inhibits cofilin oligomerization *in vitro*

**Figure 4:**
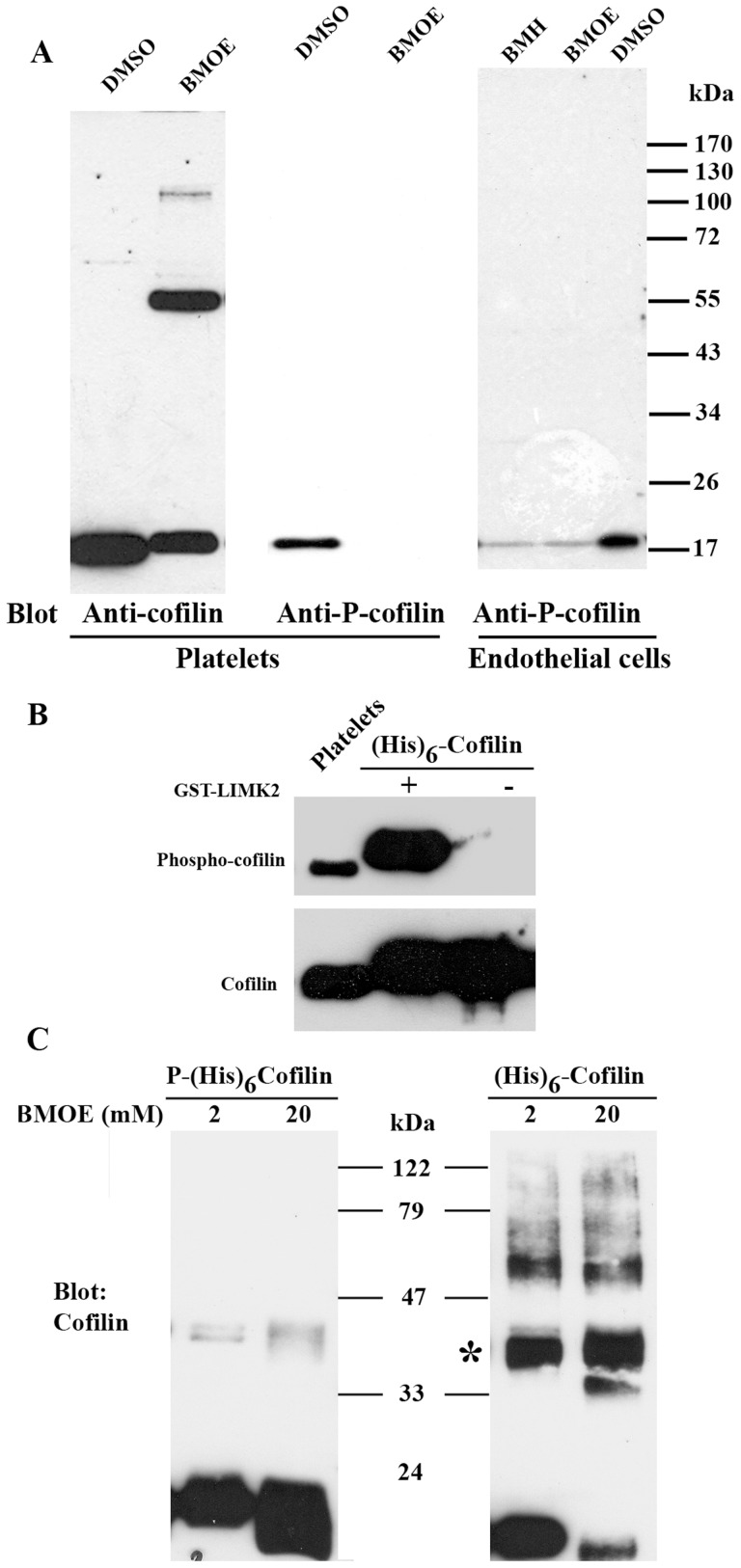
Phospho-cofilin is absent in the cofilin oligomer, and cofilin phosphorylation at Ser-3 inhibits cofilin oligomerization *in vitro*. A) The cross-linked platelet and endothelial cells lysates (as described in Figure 1A) were immunoblotted with anti-cofilin or anti-phospho-cofilin antibody. Only cofilin but not phosphorylated cofilin could be detected in the BMOE-cross-linked cofilin oligomer. B) Recombinant His-tagged cofilin was phosphorylated by GST-LIMK2 *in vitro*. The phosphorylation of cofilin was confirmed by immunoblotting using anti-phospho-cofilin antibody (upper panel). The total amount of cofilin was assayed with an anti-cofilin antibody (lower panel). C) Phosphorylated (left panel) and unphosphorylated (right panel) cofilin (16 μg) were cross-linked with two concentrations (2 and 20 mM) of BMOE. Phospho-cofilin in contrast to cofilin was barely cross-linked by BMOE (right panel; asterisk).

The reversible phosphorylation of Ser-3 residue by LIMKs is a well-established regulatory mechanism of cofilin function; it mainly inhibits the interaction of cofilin with actin [9]. Since cofilin was found to be present in platelets and endothelial cells as a monomer as well as an oligomer, we hypothesized that the equilibrium between the two states of cofilin could be regulated by phosphorylation. To explore this we blotted *in vivo* cross-linked cell lysates of platelets and endothelial cells with a specific anti-phospho-cofilin antibody. We could not detect phospho-cofilin in the cofilin oligomer; cofilin phosphorylation was detected only in the cofilin monomer (Figure 4A). In addition, phospho-cofilin in the monomeric cofilin was absent in platelets and drastically reduced in endothelial cells after cross-linking, and we could not observe any phospho-cofilin in cross-linked products of higher molecular weight. We explain these results that after phosphorylation, cofilin might interact with specific proteins such as 14-3-3ζ (see above and Discussion), which is cross-linked in very high molecular weight complexes that cannot enter the 10% gel. It is unlikely that cross-linkers may inactivate LIMK, since the cross-linkers were added to platelets after their lysis, and the lysates were kept on ice.

To directly show that phosphorylation of cofilin inhibits cofilin oligomerization, we first phosphorylated His-tagged cofilin at Ser-3 by recombinant LIMK2 *in vitro* (Figure 4B) and then cross-linked the recombinant phospho-cofilin *in vitro* by BMOE. We found that cofilin oligomer formation (e.g., dimer and trimer) was drastically reduced after phosphorylation (compare Figure 4C left and right panels). These data suggest that phosphorylation of cofilin at Ser-3 regulates the equilibrium of cofilin monomers to cofilin oligomers *in vitro*.

In order to obtain more direct evidence that phosphorylation of cofilin regulates oligomerization in endothelial cells *in vivo*, we transfected endothelial cells with cofilin-EGFP and the phospho-mimetic mutant cofilin-S3D-EGFP, subjected the cells to DMSO (control) or BMOE cross-linking, and performed immunoblotting experiments with anti-EGFP and anti-cofilin antibodies. A band of ∼100 kDa could be identified by both anti-EGFP and anti-cofilin antibodies (see arrow Figure S3) in cofilin-EGFP and cofilin-S3D-EGFP transfected cells only after BMOE cross-linking. The 100 kDa band could represent the cross-linked dimer of cofilin-EGFP (Mw about 45 kDa). We could not detect cross-linked tetramers of cofilin-EGFP (assumed Mw about 180–200 kDa) due to the smear of high molecular weight proteins cross-linked to cofilin (Figure S3). Thus we could not obtain evidence for the formation of a cofilin-EGFP tetramer (which would correspond to the cross-linked 65 kDa cofilin oligomer) in cofilin-EGFP transfected cells, and could also not analyze the effect of the phospho-mimetic mutant cofilin-S3D on cofilin-EGFP tetramer formation.

### The Rho-kinase/LIMK pathway regulates cofilin phosphorylation and cofilin aggregate formation in endothelial cells

**Figure 5:**
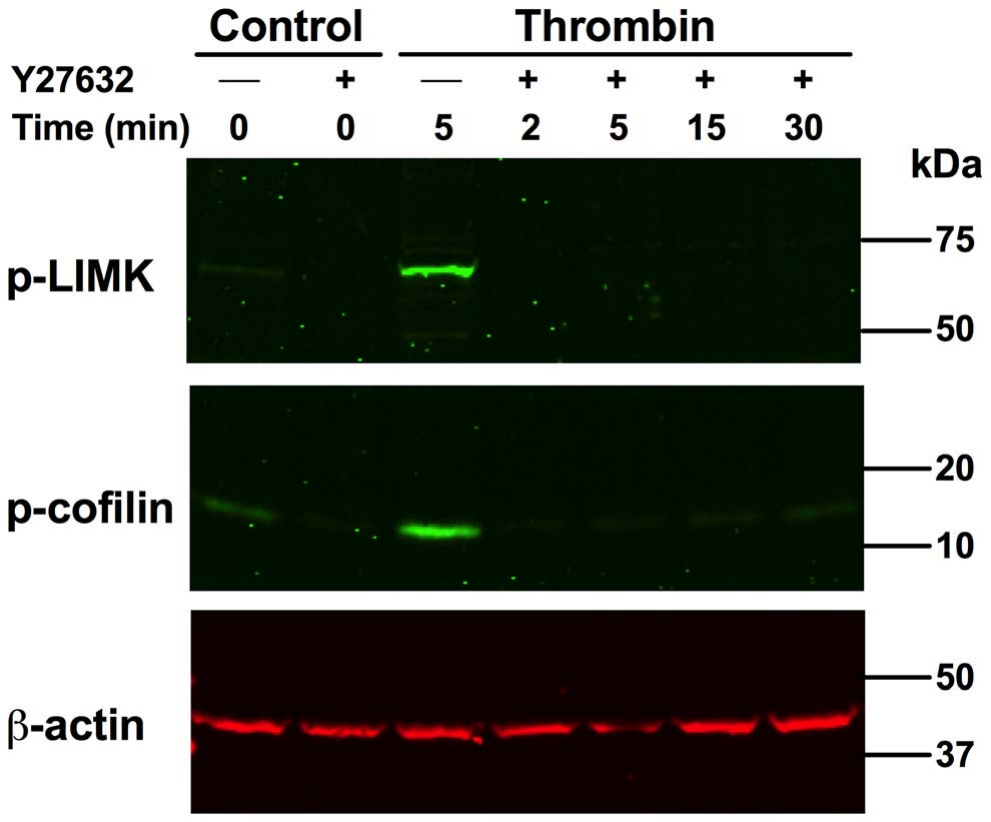
Thrombin stimulates Rho-kinase-dependent phosphorylation of LIMK and cofilin phosphorylation in endothelial cells. Endothelial cells pre-incubated without or with Rho-kinase inhibitor Y27632 (20 µM; 45 minutes) were stimulated with thrombin (1 U/ml) for different periods of time. Endothelial cells were lysed in Laemmli buffer and subjected to immunoblotting. Blots were simultaneously incubated with anti-phospho-LIMK (p-LIMK), anti-phospho-cofilin (p-Cofilin) and anti-actin antibodies.

To analyze the activation of the Rho-kinase/LIMK/cofilin pathway in thrombin-stimulated endothelial cells, phosphorylation of LIMK-kinase and cofilin was measured by specific antibodies. After thrombin stimulation of endothelial cells, LIM-kinase and cofilin were rapidly phosphorylated within 5 minutes after stimulation (Figure 5). LIMK and cofilin phosphorylation were completely inhibited by pretreatment with the specific Rho-kinase inhibitor Y27632 (Figure 5). These results show that in thrombin-stimulated endothelial cells, Rho-kinase activates LIM-kinase, which phosphorylates cofilin.

**Figure 6:**
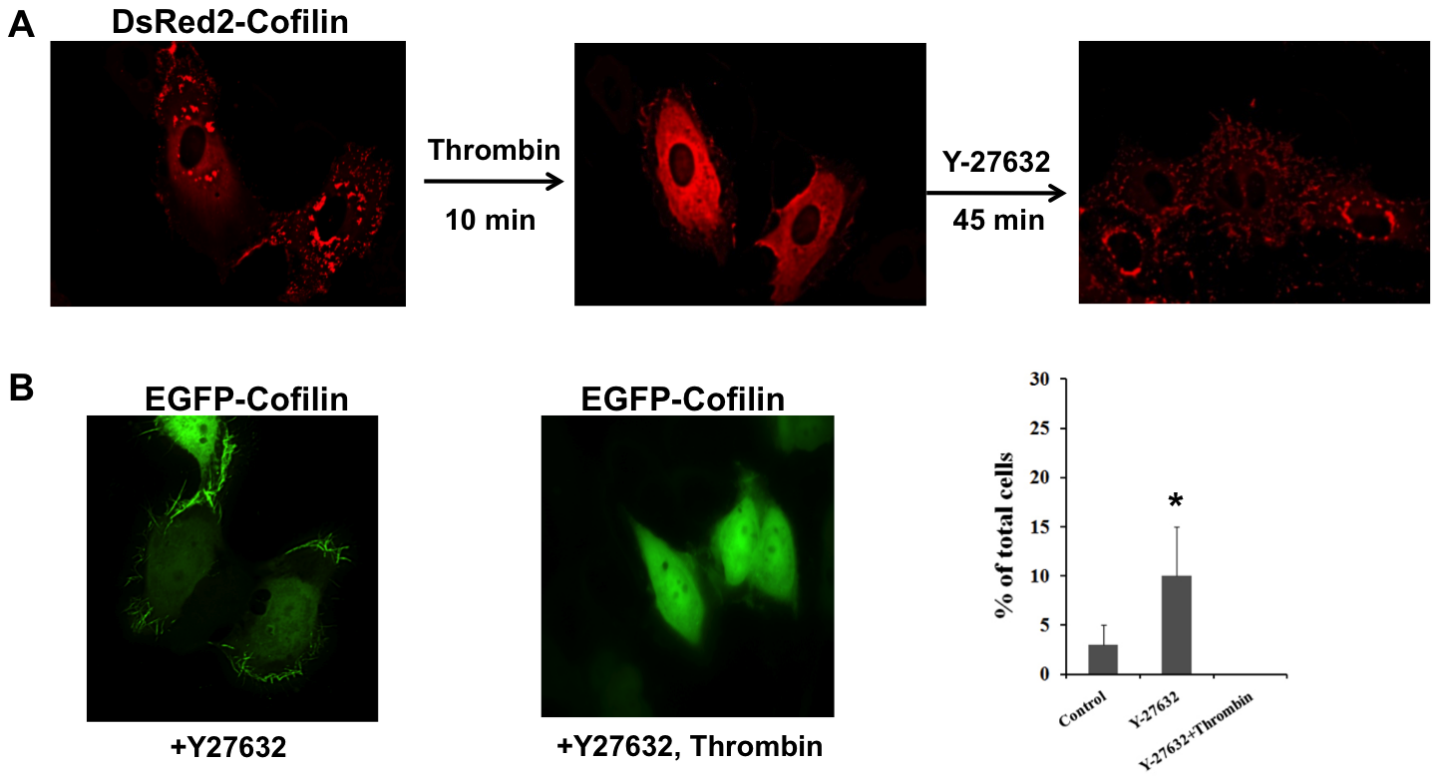
Cofilin phosphorylation regulates cofilin aggregate formation in endothelial cells. A) Endothelial cells were transfected with cofilin-DsRed2 (left panel) and activated with thrombin (1 U/ml) for 10 minutes (middle panel). Thrombin-treated cells were then incubated with Rho-kinase inhibitor (20 µM; Y27632) for 45 minutes. Aggregates of cofilin-DsRed2 were completely dissolved after thrombin treatment (middle panel) and reappeared again after incubation with Y27632 (right panel). B) Endothelial cells transfected with cofilin-EGFP were incubated with Y27632 (20 µM) for 45 minutes. Spike-like structures of cofilin-EGFP (left panel) disappeared after subsequent stimulation of cells with thrombin for 10 minutes (middle panel). Right panel, bar diagram,% of total cells with spike-like structures of cofilin-EGFP. Values are mean ± SEM of three independent experiments; *, *p*<0.05.

Next, by transfecting endothelial cells with cofilin-DsRed2, we analyzed cofilin oligomerization and its possible regulation by phosphorylation. Cofilin clusters were observed in the cytoplasm of cofilin-DsRed2- transfected confluent endothelial cells (Figure 6A, left panel; Movie S1). Interestingly, these aggregates completely dissolved within 10 minutes after exposure to thrombin, which stimulates cofilin phosphorylation. (Figure 6A; see also Movie S1). We further incubated thrombin-treated cofilin-DsRed2 transfected endothelial cells with Y27632, which inhibits LIMK-mediated cofilin phosphorylation. We observed that within 45 minutes, small cofilin aggregates appeared that were visible as dots, homogeneously distributed throughout the cytoplasm. This data suggests that phosphorylation of cofilin regulates cofilin aggregate formation in a reversible manner in intact endothelial cells.

To rule out possible additional effects of the DsRed2 tag, we also performed experiments with cofilin-EGFP. In contrast to DsRed2-cofilin, we observed in non-stimulated endothelial cells a homogenous distribution of cofilin-EGFP in the cytoplasm and the nucleus. In about 2% of the cells, we could detect green spike-like structures (Figure 6B). After incubation with Y27632, the number of cells showing these structures increased to 10% (Figure 6B). Cofilin-EGFP spikes were mainly localized near the plasma membrane (Figure 6B). Notably, after thrombin stimulation, these spike-like structures disappeared completely (Figure 6B).

In order to study whether the observed formation of cofilin aggregates in endothelial cells was due to the fluorescence tag, we transfected endothelial cells with both cofilin-EGFP and DsRed2-cofilin. We observed that cofilin-EGFP colocalized with cofilin-DsRed2in clusters indicating that cofilin forms aggregates independent of the fluorescence tag (data not shown).

We conclude that LIMK-mediated cofilin phosphorylation after thrombin stimulation of endothelial cells inhibits and dissolves cofilin aggregate formations. In contrast, inhibition of LIMK-mediated cofilin phosphorylation promotes cofilin aggregate formation in endothelial cells. The regulation of cofilin aggregate formation by phosphorylation is a reversible process.

### Cofilin dephosphorylation stimulates cofilin oligomer formation in thrombin-stimulated platelets

**Figure 7:**
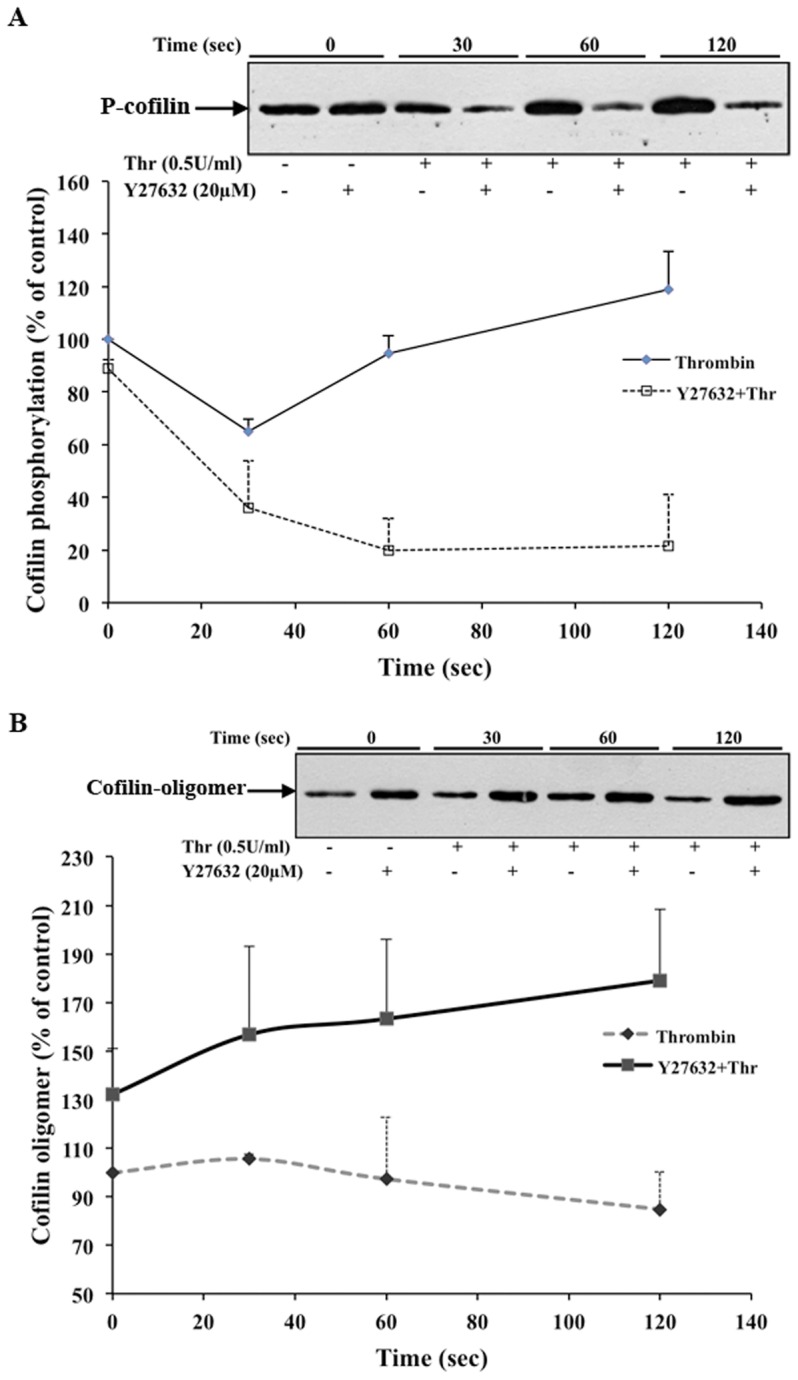
Cofilin dephosphorylation stimulates cofilin oligomer formation in platelets. Effects of Y27632-treatment and thrombin stimulation. Human washed platelets were incubated with Y27632 (20 µM) or solvent (water) for 20 minutes and then stimulated by thrombin (0.5 U/ml). Platelets were lysed in Laemmli buffer for immunoblot analysis with anti-phospho-cofilin antibody or with lysis buffer containing BMOE (0.1 mM) for immunoblot analysis of cofilin oligomer with anti-cofilin antibody. A) Graphic representation of changes of cofilin phosphorylation in thrombin-stimulated platelets in the presence or absence of Y27632. Values are the mean ± SD for four independent experiments. Insert: representative anti-phospho cofilin immunoblot. B) Graphic representation of changes of cofilin oligomer (65 kDa) formation in thrombin-stimulated platelets in the presence or absence of Y27632. Values are the mean ± SD for four independent experiments. Insert: representative immunoblot of cofilin oligomer (65 kDa) formation.

Previously, we have demonstrated that cofilin is reversibly dephosphorylated in thrombin-stimulated platelets [12]. Based on our findings in endothelial cells, we hypothesized that cofilin dephosphorylation might lead to an increased formation of the cofilin oligomer in thrombin-stimulated platelets. In agreement with our earlier study, cofilin showed a maximal (∼35%) dephosphorylation at 30 sec after platelet stimulation with thrombin (Figure 7A). The amount of the cofilin oligomer slightly, but significantly increased 30 sec after thrombin-stimulation (*p*<0.05) (Figure 7B). Pretreatment of platelets with Y27632, leading to a decrease in cofilin phosphorylation in resting platelets and to the inhibition of the cofilin rephosphorylation step in thrombin-stimulated platelets [Figure 7; [12]], was associated with a 30% increase in the amount of cofilin oligomer formation in resting platelets (Figure 7B), and a further increase of up to 70% at 2 min after thrombin stimulation, which correlates with a pronounced cofilin dephosphorylation (80%) at this time point (Figure 7). These observations suggest that cofilin dephosphorylation regulates the formation of the cofilin oligomer in platelets.

## Discussion

In the present study, we have found that cofilin exists as a monomer and 65 kDa oligomer in endothelial cells and platelets. LIMK-mediated phosphorylation of cofilin at Ser-3 inhibited the formation of the cofilin oligomer *in vivo* and also *in vitro,* whereas cofilin dephosphorylation enhanced the cofilin oligomer formation.

*In vitro* chemical cross-linking has been widely used to study protein-protein interactions. In contrast, far fewer studies have been performed to study protein-protein interactions with membrane permeable cross-linkers *in vivo* [19], [20]. To detect and stabilize the possible cofilin oligomers in intact cells, we used two cysteine-based membrane permeable non-cleavable cross-linkers of different lengths (BMOE, 8 Å; BMH, 13 Å), and an amine-based reversible cross-linker (formaldehyde, 2.3–2.7Å). Human cofilin has only four cysteines. We found that the two cysteine-based cross-linkers could specifically cross-link endogenous cofilin in endothelial cells and platelets. In these cells, we found one predominant cofilin oligomer, which had a molecular weight of ∼65 kDa on SDS-PAGE. Formaldehyde is an amine-based bifunctional cross-linker, membrane permeable and contains a very short spacer arm in the range of 2.3–2.7 Å. These features allow only for very specific cross-linking reactions between amino acids on two proteins found in very close proximity like native protein complexes found in cells. Formaldehyde freezes protein-complexes rapidly in cells and is a reversible cross-linker with MS compatibility [21], [22]. The same 65 kDa cofilin oligomer was found after formaldehyde cross-linking. We suggest that the cofilin oligomer is a cofilin tetramer (4×17 kDa), which due to cross-linking, shows a slightly lower apparent molecular weight [20]. In contrast to the *in vivo* cross-linking studies, we found that *in vitro* cross-linked recombinant cofilin exists as dimer, tetramer, and higher molecular weight oligomers. These findings are in agreement with the results of a previous study [14].

An interesting question is, why a cofilin dimer was not detectable in intact cells. We could, however, obtain some evidence for the formation of a cofilin-EGFP dimer in transfected endothelial cells (Fig. S3). A possible explanation could be that *in vivo*, oligomerization of endogenous cofilin is highly regulated to perform specific cell functions. Cofilin dimer formation *in vivo* might be a transient stage with a very short half-life that forms stable cofilin tetramers. The cofilin tetramer might be the functional unit of the cofilin oligomer.

To cross-link the cofilin oligomer by the BMOE probe *in vivo*, a minimum of two cysteine residues must be present as a free sulfhydryl, and the distance between the free cysteine residues should not be greater than 8 Å. Our data suggests that at least two cysteine residues are present on the protein-protein interacting surface of the cofilin oligomer *in vivo*. Previously, it has been shown that cofilin oligomerizes *in vitro* through intermolecular disulphide bonds that involved Cys39 and Cys147 [14]. Disulphide-linked oligomers of proteins remain intact and can be detected by SDS-PAGE analysis under non-reducing conditions [23], [24]. Our results show that intermolecular disulphide bonds are not involved in the formation of cofilin oligomers in intact endothelial cells and platelets; cofilin oligomer formation was not observed after SDS-PAGE separation of platelet and endothelial cell lysates under non-reducing conditions.

It was not possible for us to observe cofilin oligomers *in vivo* without stabilizing the oligomers with chemical cross-linkers. However, it has been clearly shown by us and others that *in vitro* cofilin can form dimers, and high MW oligomers [14]; Figure 2B). This is dependent on the concentration of cofilin in solution. Cofilin oligomer formation *in vitro* is stabilized by S-S bonds [14]; Figure 2B). By MS analysis of cross-linked His-tagged cofilin, we found, in agreement a with a previous report [14], that Cys147 was not modified by iodacetamide suggesting that Cys147 was not free, and is therefore involved in intermolecular disulphide bond formation of recombinant cofilin (data not shown). In contrast to the *in vitro* results, S-S bonds are not involved in cofilin oligomer formation *in vivo*: No cofilin oligomers are detected after cell lysis and protein separation under non-reducing conditions.

We found that monomeric cofilin (endogenous and recombinant His-tagged) had a higher electrophoretic mobility using SDS-PAGE under non-reducing conditions, as compared to reducing conditions, similar to other disulphide bond containing proteins reported previously [25]–[27]. This suggests that intramolecular disulphide bonds are already present in cofilin under physiological conditions that did not disturb the ability of cofilin to be phosphorylated by LIMKs. In our study, both His-tagged cofilin and endogenous cofilin likely to contain intra-molecular disulphide bonds were able to be phosphorylated by LIMK *in vitro* and *in vivo,* respectively. This is in contrast to a previous study showing that oxidation of cofilin induced the formation of an intramolecular disulfide bond and an inability of cofilin to be phosphorylated at Ser-3 [17].

As already mentioned, human ADF and cofilin both show 72% sequence homology. Interestingly, ADF is a more potent actin-depolymerizing protein than cofilin [15], [16]; in our study, ADF showed a much weaker tendency to form the ∼65 kDa oligomer in platelets and endothelial cells than cofilin. Notably, ADF has eight cysteine residues but only three (C39, C80, and C147) are at the same positions as in cofilin (C39, C80, and C147) (Figure S1). ADF has a cysteine residue at position 135 instead of position 139, which is present in cofilin. The largest sequence difference of cofilin and ADF is in the C-terminal region (aa 139–165) (Figure S1). We, therefore, suggest that the C-terminal part, especially the region of Cys139 of cofilin, plays an important role in cofilin oligomerization.

The reversible phosphorylation of Ser-3 residue by LIMKs is a well-established regulatory mechanism of cofilin function. Based on our results, we propose that an equilibrium between cofilin monomers and cofilin oligomers exists in cells, which is regulated by phosphorylation at Ser-3. We found that a) only cofilin but not phosphorylated cofilin was present in the endogenous cofilin oligomer; b) phosphorylation of recombinant His-tagged cofilin by GST-LIMK2 inhibited the formation of BMOE-cross-linked cofilin oligomers *in vitro*; and c) cofilin phosphorylation regulated the formation of cofilin aggregates in endothelial cells and cofilin oligomers in platelets *in vivo*. Cofilin modeling data (not shown) indicate that cofilin phosphorylation at Ser-3 induces conformational changes in the protein-protein interacting surface of the cofilin oligomer, thereby preventing and/or and disrupting the cofilin oligomer formation.

A band of **∼**100 kDa could be identified by both anti-EGFP and anti-cofilin antibodies (Figure S3) in cofilin-EGFP and cofilin-S3D-EGFP transfected endothelial cells after BMOE crosslinking. The 100 kDa could represent the cross-linked dimer of cofilin-EGFP, but we cannot rule out that other proteins are also present in ∼100 kDa band. Due to the high molecular weight cross-linked products smear, we could not detect any cofilin-EGFP tetramer (∼200 kDa) band. We could not find a difference of the intensity of the 100 kDa band in cross-linked cofilin-EGFP and cofilin-S3D-EGFP transfected endothelial cells. However, we think that the replacement of Ser-3 with Asp is not a good alternative to mimic the phosphorylation of serine, especially when the conformation of the protein is very critical factor for function.

In endothelial cells, cofilin at Ser-3 was phosphorylated by the Rho-kinase/LIMK pathway after thrombin stimulation. Cofilin-DsRed2 aggregates in transfected endothelial cells dissolved within 10 minutes after thrombin stimulation, indicating that phosphorylation of cofilin at Ser-3 might be responsible. Notably, after subsequently inhibiting the Rho-kinase dependent activation of LIMK, small cofilin-DsRed2 aggregates reappeared within 45 minutes. These data prompted us to suggest that phosphorylation of cofilin is the mechanism that regulates cofilin aggregate formation *in vivo* in endothelial cells in a reversible manner. In contrast to DsRed2, which exists as a dimer in cells, EGFP exists as a monomer. We observed a homogeneous distribution of cofilin-EGFP in the cytoplasm in the majority of cells, and in some cells, spike-like structures that were localized beneath the plasma membrane. After Y27632 treatment, these structures were visible in a greater number of cells. Subsequent treatment with thrombin further dissolved these structures, indicating that cofilin-phosphorylation regulates cofilin-spike formation in a reversible manner. The presence of cofilin-EGFP in cofilin-DsRed2 aggregates in co-transfected cells indicates that cofilin has a tendency of self-association. Together, these data can be explained as follows: when the local concentration of the unphosphorylated cofilin increases over a threshold value, cofilin starts to oligomerize into a tetramer, leading to cofilin aggregate formation in the case of cofilin-DsRed2, which amplifies the assembly process due to the dimerizing property of the fluorescent tag. Stimulation of cofilin phosphorylation inhibits cofilin oligomer formation and cofilin aggregate formation, whereas inhibition of cofilin phosphorylation increases cofilin oligomer formation and cofilin aggregate formation.

Previously, cofilin-actin rods were observed in various cell types under cellular stress, such as after DMSO treatment or ATP depletion [28], [29]. As the cofilin-actin heterodimer has a molecular weight of 62 kDa, we considered whether the cofilin oligomer observed *in vivo* might contain actin. However, we have shown by using different experimental approaches that it does not. This is in contrast to the results of *in vitro* cross-linking using purified actin and cofilin proteins: here the cross-linked complexes contained monomeric and oligomeric actin (Figure 3B).

Apparently the situation *in vivo* is different. It might be argued that the cofilin oligomer formation might only occur when cofilin is bound to F-actin (thus resulting in the exclusion of phospho-cofilin from the complexes). Cofilin binds F-actin cooperatively and could, at the concentrations where cross-linking occurs, bind and saturate small regions of F-actin leading to the 65 kDa cofilin tetramer. However, if this is the case, actin should also be cross-linked with the cofilin oligomer, which is not the case (see above). Moreover, the distance between cofilin molecules on actin fibers is much higher than the 8Å and 2.3–2.7 Å distances required for protein cross-linking by BMOE and formaldehyde, respectively, suggesting that the 65 kDa tetramer formation on F-actin is not possible [30].

Besides actin, other proteins such as Memo (33 kDa), Aip1 (65 kDa), Cap1 (51.67 kDa) and Nck1 (42.86 kDa) were reported to directly interact with cofilin or present in the complex of actin and cofilin [5], [31]. The theoretical molecular weight of complexes of cofilin with these proteins (except Nck1) is different of the ∼65 kDa oligomer. We have addressed the question whether the cofilin oligomer contains Nck1 by doing immunoblotting of cross-linked endothelial cells lysates. We could not detect Nck1 protein in the 65 kDa cofilin oligomer, although we found that endothelial cells express Nck1 (data not shown). Also Nck1 was not found by MS analysis of cofilin immunoprecipitates after formaldehyde cross-linking of endothelial cells. In contrast the protein 14-3-3ζ (28 kDa) was detected by MS analysis in the cofilin immunoprecipitates of the whole lysates after formaldehyde cross-linking of endothelial cells. However, 14-3-3ζ is known to interact with phospho-cofilin only [18], which was not present in the cofilin oligomer. Therefore, we consider it as very unlikely that 14-3-3ζ is part of the 65 kDa cofilin oligomer.

To further support our findings in endothelial cells, we studied platelets, where we quantified the formation of the 65 kDa cofilin oligomer. We have shown in an earlier study that cofilin is maximally dephosphorylated 30 sec after thrombin stimulation of platelets [12]. Furthermore, we had found that the rapid dephosphorylation of cofilin is regulated via Rac1 mediated class II PAKs activation [13]. In the present study, we found that cofilin oligomer formation significantly increased at this time point. We further observed that the amount of cofilin oligomer also increased (30%) in Y27632-treated resting platelets, which inhibits cofilin phosphorylation. The cofilin oligomer formation increased further up to 70% in Y27632-treated cells stimulated for 2 minutes with thrombin, which caused a sustained large decrease of cofilin phosphorylation (80%). These data indicate that cofilin dephosphorylation increases the amount of cofilin oligomer in stimulated platelets. Together with our previous studies [12], [13], we propose that cofilin dephosphorylation in platelets either by activation of a Rac1 mediated pathway after thrombin-stimulation or by inhibition of LIMK by Y27632 treatment leads to cofilin oligomer formation which could explain the increase of F-actin and of the amount of cofilin associated with F-actin observed under these conditions previously [13].

**Figure 8:**
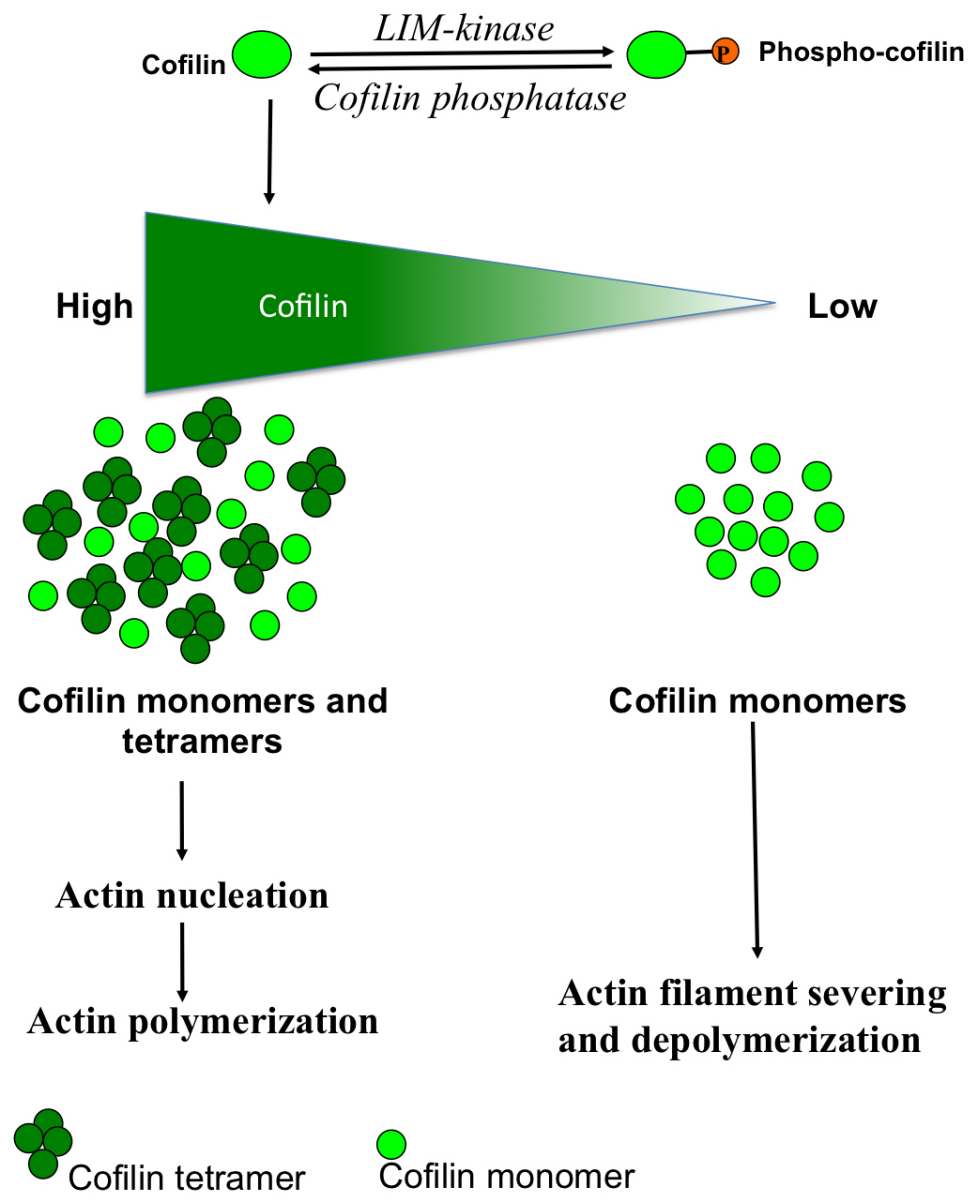
A model explaining how cofilin phosphorylation might regulate cofilin oligomer formation, and thereby its dual function on actin dynamics.

Based on our results and previous studies, we propose the following model of regulation of cofilin function (Figure 8). Cells contain cofilin monomers and oligomers, which are in equilibrium. This regulates the cofilin-dependent actin dynamics in the direction of either actin polymerization (cofilin oligomer) or actin depolymerization (cofilin monomer). It has been shown previously that cofilin oligomers formed *in vitro* increase actin polymerization [14]. Phosphorylation and dephosphorylation of cofilin at Ser-3 may influence the cofilin monomer/oligomer equilibrium and thereby regulate the dual activity of cofilin. Local dephosphorylation of cofilin might increase the pool of oligomeric cofilin at the account of monomeric cofilin, leading to high local concentrations of the cofilin tetramer and increase actin polymerization (Figure 8). The region containing Cys139 exposed on the surface of the cofilin monomer is likely to be present on the protein-protein interacting surface of the cofilin oligomer. Rho-kinase/LIMK-dependent phosphorylation of cofilin at Ser-3 induces a conformational change of cofilin and promotes cofilin monomer formation. Dephosphorylation of monomeric coflin at low local concentrations promotes actin depolymerisation. The local cofilin concentration above a certain threshold value induces cofilin-cofilin association, which is regulated in such a manner that only one type of cofilin oligomer (tetramer) can form.

## Materials and Methods

### Construction of the Expression Plasmids

To attach the reporter gene (*EGFP or DsRed2*) at the 3′ end of the *cofilin gene*, the full-length coding sequence of cofilin was amplified by PCR from a cDNA pool of human umbilical vein endothelial cells (HUVEC) and then cloned into the XhoI and BamHI sites of the pEGFP-N1, pDsRed2-N1 vector (Clontech Inc). A phospho-mimetic mutant (cofilin-S3D-EGFP) was prepared by using Quick-Change II site–directed mutagenesis Kit (Stratagene, Agilent Technologies) as per manufacturer’s instructions. To prepare FLAG-tagged cofilin plasmid, *EGFP* gene was deleted and FLAG tag (DYKDDDDK) coding oligonucleotide was inserted in cofilin-EGFP plasmid by using Quick-Change II site-directed mutagenesis kit. To prepare His-tagged cofilin (cofilin-(His)_6_) construct, cDNA of cofilin was cloned into the BamH1 and NcoI sites of the pQE60 vector (Qiagen GmbH).

### Endothelial cell culture

HUVECs were obtained and cultured as described previously [32]. Briefly, HUVECs harvested from umbilical cords were plated onto collagen-coated plastic culture flasks and were cultured at 5% CO_2_ and at 37°C in complete endothelial growth medium (Promo Cell, Germany).

### *In vivo* cross-linking of cofilin in endothelial cells with BMOE or BMH

Confluent endothelial cells were treated with trypsin and EDTA to prepare a cell suspension. The cells were pelleted by centrifugation, resuspended in 0.5 ml of complete endothelial growth medium, and incubated on ice for 30 minutes. Endothelial cells were distributed in sterile polypropylene tubes at a density of approx. 1.0×10^6^ cells in 20 µl of complete medium. Cross-linker (BMOE or BMH, 1 mM; Thermo Fisher Scientific Inc.) or solvent (DMSO) was added to the cells and then incubated at 37 °C for one hour in a CO_2_ cell incubator. The cross-linking reaction was stopped by adding 2× Laemmli buffer and subjected to immunoblotting.

### In vivo cross-linking of cofilin in endothelial cells with formaldehyde

HUVECs in suspension (5×10^6^) were centrifuged at 100 x g for 5 minutes. Cell pellet was resuspended in 4.5 ml complete endothelial growth medium. Formaldehyde was used as cross-linking agent based on a previous study [33]. In brief, formaldehyde (1% final concentration) was added to the cells and then incubated for 10 minutes at room temperature on an end-to-end-shaker. The cross-linking reaction was stopped by adding glycine (125 mM final concentration), and incubation for 15 minutes at room temperature while rotating. The cells were pelleted by centrifugation at 4 °C and then were subjected for Western blotting or immunoprecipitation.

### Isolation of human platelets and *in vivo* cross-linking of cofilin in platelets

Blood was taken from human volunteers. Approval was obtained from the Ethic Commission of the Medical Faculty of the University of Munich. Informed consent was provided according to the Declaration of Helsinki. Platelets from acetylsalicylic acid–treated human blood were isolated as described previously [12]. Human washed platelet suspensions (4×10^8^/ml, 0.4 ml) were stimulated with thrombin (Sigma) in the presence or absence of the Rho-kinase inhibitor Y27632 (Calbiochem, Merck Biosciences GmbH). RGDS (Bachem Biochemica, Germany) was added prior to thrombin-stimulation in order to avoid platelet aggregation. Aliquots (100 µl) of unstimulated and stimulated platelet suspensions were lysed in an equal volume of ice cold 2x lysis buffer (50 mM HEPES, pH 7.5, 300 mM NaCl, 2% NP-40, 20 mM MgCl_2_, 2 mM EDTA, 4% glycerol, 5 mM Na_3_VO_4_, phosphatase cocktail 1∶100, and complete mini protease inhibitor 1 tablet/8 ml) with or without the chemical cross-linker BMOE or BMH (0.2 mM) for 20 minutes on ice. The lysates were then transferred to an equal volume of 2x SDS-PAGE sample buffer for immunoblotting.

### Western blotting and measurement of changes in protein by densitometry

Western blotting was done as described previously [32] using anti-ß-actin (1∶100,000; Cytoskeleton Inc), anti-cofilin (1∶10,000; (Cytoskeleton Inc), anti-ß-phospho-cofilin antibody (1∶1000; Cell Signaling Technology), anti-phospho-LIMK antibody (1∶1000; Cell Signaling Technology), anti-FLAG-M2 peroxidase conjugate antibody (1∶3000; Sigma), anti-EGFP antibody (1∶1000; Clontech, Takara Bio company) and anti-ADF (1∶1000; Sigma) as primary antibodies.

Densitometric analysis of the proteins was performed using the public domain NIH ImageJ (version 1.32j) software. The films were scanned into TIF format using a ScanJet 5300C (Hewlett-Packard). The optical density of proteins in unstimulated control samples was set to 100%. Data are presented as mean ±S.E. of three independent experiments.

In later stages of the study we used Odyssey Infrared Imager for scanning the membranes. Briefly, after primary antibody incubation, the secondary goat-anti-rabbit 800 antibody and goat-anti-mouse 680 antibody (LI-COR Biosciences GmbH, Bad Homburg, Germany) were used with a dilution of 1:10,000. Protein signal was detected using the Odyssey Infrared Imager with application Software 3.0.30 (LI-COR Biosciences).

### Expression and purification of recombinant His-tagged human cofilin

The pQE-60-cofilin plasmid was transformed in M15 *E.coli* cells (Qiagen) and grown at 37 °C. The expression of recombinant cofilin was induced by adding 1 mM isopropyl ß-d-thiogalactoside at an *A*_600_ of ∼0.6 for three hours. Bacteria were harvested by centrifugation and resuspended in 10 ml of ice-cold lysis buffer (PBS buffer containing complete mini protease inhibitors (Roche Diagnostics), 10 mM imidazole, lysozyme (1 µg/ml); pH 8.0) and incubated on ice for 30 minutes. The bacterial cells were lysed by sonication, and then the lysate was incubated with DNAse I (5 µg/ml) on ice for 15 minutes. Cell debris was removed by centrifugation at 60,000xg, and the supernatant was loaded on Ni-NTA Superflow column (GE lifesciences) pre-equilibrated with lysis buffer. The column was then washed thoroughly with lysis buffer and then bound His-tagged cofilin was eluted with elution buffer (PBS buffer containing 250 mM imidazole; pH 8.0). Eluted fractions containing high amounts of purified His-tagged proteins (>97%) were selected, pooled, desalted, and concentrated using Centricon® Plus-20 (Millipore).

### *In vitro* phosphorylation of cofilin by recombinant LIMK2

GST beads (GE Life sciences) were washed twice with kinase buffer (50 mM HEPES, 5 mM MgCl2, 10 mM NaF, 1 mM Na3VO4, and pH 7.5) and then used as 50% slurry to couple LIMK2. GST-LIMK2 (5 µl, Invitrogen) was incubated with 30 µl of a 50% GST-bead slurry in a volume of 100 µl kinase buffer at 30°C for one hour. LIMK2 bound GST beads were pelleted at 13,000 rpm for 15 sec, washed once with kinase buffer, and resuspended in 100 µl kinase buffer containing ATP (10 mM). His-tagged cofilin (10 µl; 1 mg/ml in PBS) were added in kinase buffer with GST-LIMK2, and the reaction mixture was incubated for 1 hour at 37 °C. The GST-LIMK2 beads were pelleted by centrifugation at 13,000 rpm for 15 sec, and the supernatant containing phosphorylated His-tagged cofilin was transferred to a new tube and subjected to *in vitro* cross-linking with BMOE.

### *In vitro* cross-linking of cofilin and cofilin with actin

Different concentrations of the purified recombinant human His-tagged cofilin and phosphorylated His-tagged cofilin or equal concentrations of actin (Cytoskeleton Inc.) and human cofilin (without His-tag) were taken in reaction buffer (PBS; pH 7.2) with a final volume of 50 µl. Cross-linker (BMOE or BMH, 20 mM stock; two molar excess of protein) or solvent (DMSO) were added in reaction buffer and incubated one hour at room temperature. The reaction was stopped by adding 2x Laemmli buffer and subjected to SDS-PAGE.

### Immunoprecipitation of cofilin after cross-linking of cells with BMOE

Immunoprecipitation of cross-linked cofilin oligomers and cofilin-protein complexes was performed by using the method for immunoprecipitation of cross-linked proteins described previously [19]. Briefly, endothelial cells treated with the cross-linkers BMOE were pelleted and resuspended in 1.5 ml PBS containing 5% ß-mercaptoethanol (ßM), which quenched any residual BMOE. Cells were pelleted again and resuspended in 0.1 ml of PBS. Platelet lysates treated with BMOE were also quenched with 5% ßM. Guanidine hydrochloride (GuHCI; 4 volumes of 8 M) containing 2 mM DTT was added to the cell suspensions and platelet lysates. This mixture was homogenized through a 22-gauge needle and heated at 95 °C for 5 min. Samples were diluted in IP buffer (20 mM Hepes (pH 7.5), 10% glycerol, 0.1 mM EDTA, 2 mM MgCI_2_, 0.1 mM DTT, and 0.2 mM phenylmethylsulfonyl fluoride) to a final GuHCI concentration of 1 M. Cell debris were removed by centrifugation at 14,000xg for 15 min at 4 °C, and then 150 µl of 50% protein A–Sepharose slurry was added to the supernatants and incubated for 1 hour at 4 °C to preclear the supernatant. Protein A–Sepharose was prepared by incubating the beads in swelling buffer (20 mM Hepes (pH 7.5), 0.15 M NaCl, and 0.1% NaN_3_) containing 2% BSA to block unspecific binding. Precleared supernatants were incubated overnight either with 80 µl of anti-FLAG-M2 gel-slurry (endothelial cell lysates) or anti-cofilin antibody (1∶100 dilution) (platelet lysates). The next day, 80 µl of 50% protein A–Sepharose slurry was added to the supernatant containing the anti-cofilin antibody followed by incubation at 4 °C for two hours. The beads were pelleted by centrifugation and washed twice with IP buffer containing 1 M GuHCI and 0.005% nonidet-40, followed by a wash with IP buffer lacking GuHCI. Finally, beads were resuspended in 100 µl 1x Laemmli buffer, boiled for 5 minutes at 95 °C, and subjected to SDS-PAGE and western blotting.

### Immunoprecipitation of cofilin of formaldehyde-cross-linked endothelial cells for mass spectroscopy

The cell pellets after formaldehyde cross-linking were resuspended in 200 µl RIPA buffer (Thermo Scientific Inc, MA, USA) for one hour at 4°C. Cell lysates were homogenized with 22-gauge needle 30 minutes after resuspension in RIPA buffer. Cell lysates were then centrifuged at 16,000 x g for 30 minutes at 4°C, and the supernatant was used for further analysis. For cofilin immunoprecipitation, anti-cofilin antibody (1 100 dilution) was added to the supernatant and incubated overnight at 4 °C. Protein A magnetic beads (25 µl; New England Biolab GmbH) were added and incubated for one hour. The beads were magnetically removed, washed thrice with 500 µl IP wash buffer (Active Motif, La Huple, Belgium), and then resuspended in 50 µl PBS. For control, protein A magnetic beads (25 µl) was added to the supernatant, and it was proceeded as described above. Control and cofilin antibody bounds proteins were subjected to mass spectroscopy.

### Mass spectrometric analysis of cofilin interacting proteins

Beads with control and cofilin antibody bound proteins were washed three times with 100 µl ammonium bicarbonate (20 mM) before proteolytic digestion with trypsin (300 ng) at room temperature under rotation for 16 h. The reaction was stopped with a final concentration of 1% acetic acid and the peptide mixtures were desalted on C-18 reverse phase material (ZipTip µ-C18 Millipore Corporation, MA, USA) and further processed as described earlier [34]. Briefly, peptides were eluted in 50% and 80% acetonitrile (ACN) each in 1% acetic acid. Pooled eluates were concentrated to 2 µl in a vacuum concentrator (5303, Eppendorf, Wesseling, Germany) and resuspended in 2% ACN in 0.1% acetic acid. Peptide separation was performed using a non-linear 86 min gradient of 5–40% ACN in 0.1% acetic acid at a constant flow rate of 300 nl/min on a reverse phase column (PepMap C18, 75 µm id x 15 cm, LC Packings, Idstein, Germany) operated on an Easy- nLC (Proxeon, Thermo Scientific, Dreieich, Germany). The eluate was directed into a LTQ-Orbitrap Velos mass spectrometer (Thermo Electron, Bremen, Germany) and data dependent 20 MS/MS spectra were recorded. The experiments were performed three times.

### Protein identification

Proteins were identified via automated database search in a forward-reverse Uniprot database (rel. 01/2012) limited to human entries using the Sequest algorithm rel. 2.7 (Sorcerer built 4.04, Sage-N Research Inc., Milpitas, CA, U.S.A.). Parent mass tolerance (MS) was set to 10 ppm and fragment mass tolerance 1 Da. Methionine oxidation was considered as optional modification. Proteins and peptides were considered as significantly identified when probability was >95%, respectively (Peptide and Protein Prophet, Scaffold rel. 3.4, Proteome Software, Portland, OR, USA).

### Transfection of endothelial cells

In all experiments, HUVECs were transfected with 2 µg DNA per 1×10^6^ cells using the HUVEC nucleofactor kit from Amaxa GmbH.

### Fluorescence microscopy

After 24h of transfection, cells were observed with a Nikon TE2000E-PFS fluorescence microscope with 37°C incubation chamber. The microscope was controlled through NIS-Elements software. The measurements were carried out in three independent experiments with 50 cells randomly selected in each experiment. The mean ± S.E. was calculated for each experiment.

### Statistical analysis

The experiments shown are representative of at least three others, which gave similar results. Values given are mean ± SEM or mean ± SD as indicated in the Figure legends. Significant difference was determined by the paired Student’s t-test or other tests as appropriate. A p-value of <0.05 was considered statistically significant.

## Supporting Information

Figure S1 **Protein sequence alignment of human non-muscle cofilin (CFL1-Human) and human ADF (ADF-Human).** Cysteine residues are highlighted in yellow. The positions of cysteine in human cofilin are indicated.
(RTF)

Figure S2 **ADF does not form a distinct 65 kDa oligomer after cross-linking of endothelial cells.** Endothelial cells (0.8–1×10^6 ^cells/20 µl) were incubated with DMSO (1 µl) or BMOE (1 mM). For formaldehyde cross-linking, endothelial cells (1×10^6 ^cells/ml) were treated with formaldehyde at a final concentration (1%). The cell lysates were subjected to SDS-PAGE on gradient gel (4–15%) and were then immunoblotted with an anti-ADF antibody. A smear of ADF cross-linked proteins was observed for both BMOE and formaldehyde cross-linked endothelial cells. Proteins were detected by fluorescence imaging of secondary antibodies labeled with infrared dyes.
(PDF)

Figure S3 **Immunoblotting of cofilin-EGFP and cofilin-S3D-EGFP** **transfected and cross-linked endothelial cells.** Endothelial cells were transfected with EGFP, cofilin-EGFP or cofilin-S3D-EGFP plasmid. After 20 hours of transfection, cells (0.8–1×10^6 ^cells/20 µl) were treated with DMSO (1 µl) or BMOE at a final concentration of 1 mM. The cell lysates were subjected to SDS-PAGE on a gradient gel (4–15%) and were then immunoblotted with anti-cofilin or anti-EGFP antibody. A band of **∼**100 kDa was apparent in both anti-EGFP and anti-cofilin immunoblots (arrow) of lysates of cofilin-EGFP and cofilin-S3D-EGFP transfected cells after BMOE cross-linking.
(PDF)

Movie S1 **Live cell imaging of human endothelial cells transfected with DsRed2-cofilin.** Endothelial cells transfected with cofilin-DsRed2 were stimulated with thrombin (1 U/ml) and then observed under the Nikon TE2000E PFS microscope with 63x magnification at 37°C. Pictures were taken every 30 seconds for 30 minutes using NIS elements software. Movie was edited with QuickTime Pro from Apple Inc.
(MOV)
