# Supplementary material for: Cofilin Oligomer Formation Occurs In Vivo and Is Regulated by Cofilin Phosphorylation
Source: PLoS One. 2013 Aug 8;8(8):e71769. doi: 10.1371/journal.pone.0071769 (PMC3738525; doi:10.1371/journal.pone.0071769)
Supplement: Figure S1 — Protein sequence alignment of human non-muscle cofilin (CFL1-Human) and human ADF (ADF-Human). Cysteine residues are highlighted in yellow. The positions of cysteine in human cofilin are indicated. (RTF) [file pone.0071769.s001.rtf]

                                                      39
CFL1-Human    1 MASGVAVSDGVIKVFNDMKVRKSSTPEEVKKRKKAVLFCLSEDKKNIILEEGKEILVGDV 60
ADF-Human     1 MASGVQVADEVCRIFYDMKVRKCSTPEEIKKRKKAVIFCLSADKKCIIVEEGKEILVGDV 60
                ***** *:* * ::* ******.*****:*******:**** *** **:***********

                                   80
CFL1-Human   61 GQTVDDPYATFVKMLPDKDCRYALYDATYETKESKKEDLVFIFWAPESAPLKSKMIYASS 120
ADF-Human    61 GVTITDPFKHFVGMLPEKDCRYALYDASFETKESRKEELMFFLWAPELAPLKSKMIYASS 120
                * *: **:  ** ***:**********::*****:**:*:*::**** ************

                                  139     147
CFL1-Human  121 KDAIKKKLTGIKHELQANCYEEVKDRCTLAEKLGGSAVISLEGKPL 166
ADF-Human   121 KDAIKKKFQGIKHECQANGPEDLN-RACIAEKLGGSLIVAFEGCPV 165
                *******: ***** ***  *::: *. :******* ::::** *


Figure S1. Protein sequence alignment of human non-muscle cofilin (CFL1-Human) and human ADF (ADF-Human). Cysteine residues are highlighted in yellow. The positions of cysteine in human cofilin are indicated.
