# Supplementary figures and images for: Cofilin Oligomer Formation Occurs In Vivo and Is Regulated by Cofilin Phosphorylation
Source: PLoS One. 2013 Aug 8;8(8):e71769. doi: 10.1371/journal.pone.0071769 (PMC3738525; doi:10.1371/journal.pone.0071769)

DMSO

Formaldehyde

BMOE

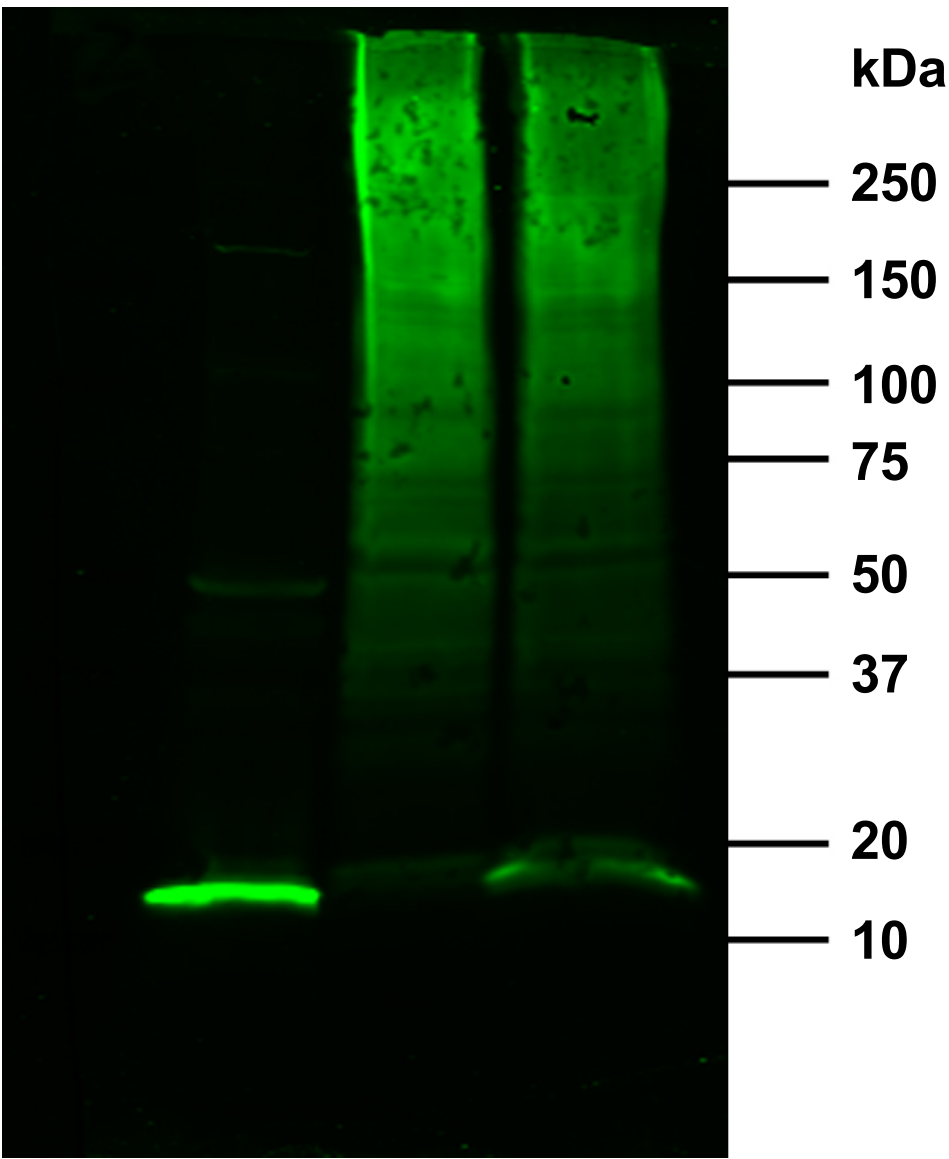

Supplement: Figure S2 — ADF does not form a distinct 65 kDa oligomer after cross-linking of endothelial cells. Endothelial cells (0.8–1×10^6^ cells/20 µl) were incubated with DMSO (1 µl) or BMOE (1 mM). For formaldehyde cross-linking, endothelial cells (1×10^6^ cells/ml) were treated with formaldehyde at a final concentration (1%). The cell lysates were subjected to SDS-PAGE on gradient gel (4–15%) and were then immunoblotted with an anti-ADF antibody. A smear of ADF cross-linked proteins was observed for both BMOE and formaldehyde cross-linked endothelial cells. Proteins were detected by fluorescence imaging of secondary antibodies labeled with infrared dyes. (PDF) [file pone.0071769.s002.pdf]
