# Supplementary material for: Cofilin Oligomer Formation Occurs In Vivo and Is Regulated by Cofilin Phosphorylation
Source: PLoS One. 2013 Aug 8;8(8):e71769. doi: 10.1371/journal.pone.0071769 (PMC3738525; doi:10.1371/journal.pone.0071769)
Supplement: Figure S3 — Immunoblotting of cofilin-EGFP and cofilin-S3D-EGFP transfected and cross-linked endothelial cells. Endothelial cells were transfected with EGFP, cofilin-EGFP or cofilin-S3D-EGFP plasmid. After 20 hours of transfection, cells (0.8–1×10^6^ cells/20 µl) were treated with DMSO (1 µl) or BMOE at a final concentration of 1 mM. The cell lysates were subjected to SDS-PAGE on a gradient gel (4–15%) and were then immunoblotted with anti-cofilin or anti-EGFP antibody. A band of ∼100 kDa was apparent in both anti-EGFP and anti-cofilin immunoblots (arrow) of lysates of cofilin-EGFP and cofilin-S3D-EGFP transfected cells after BMOE cross-linking. (PDF) [file pone.0071769.s003.pdf]

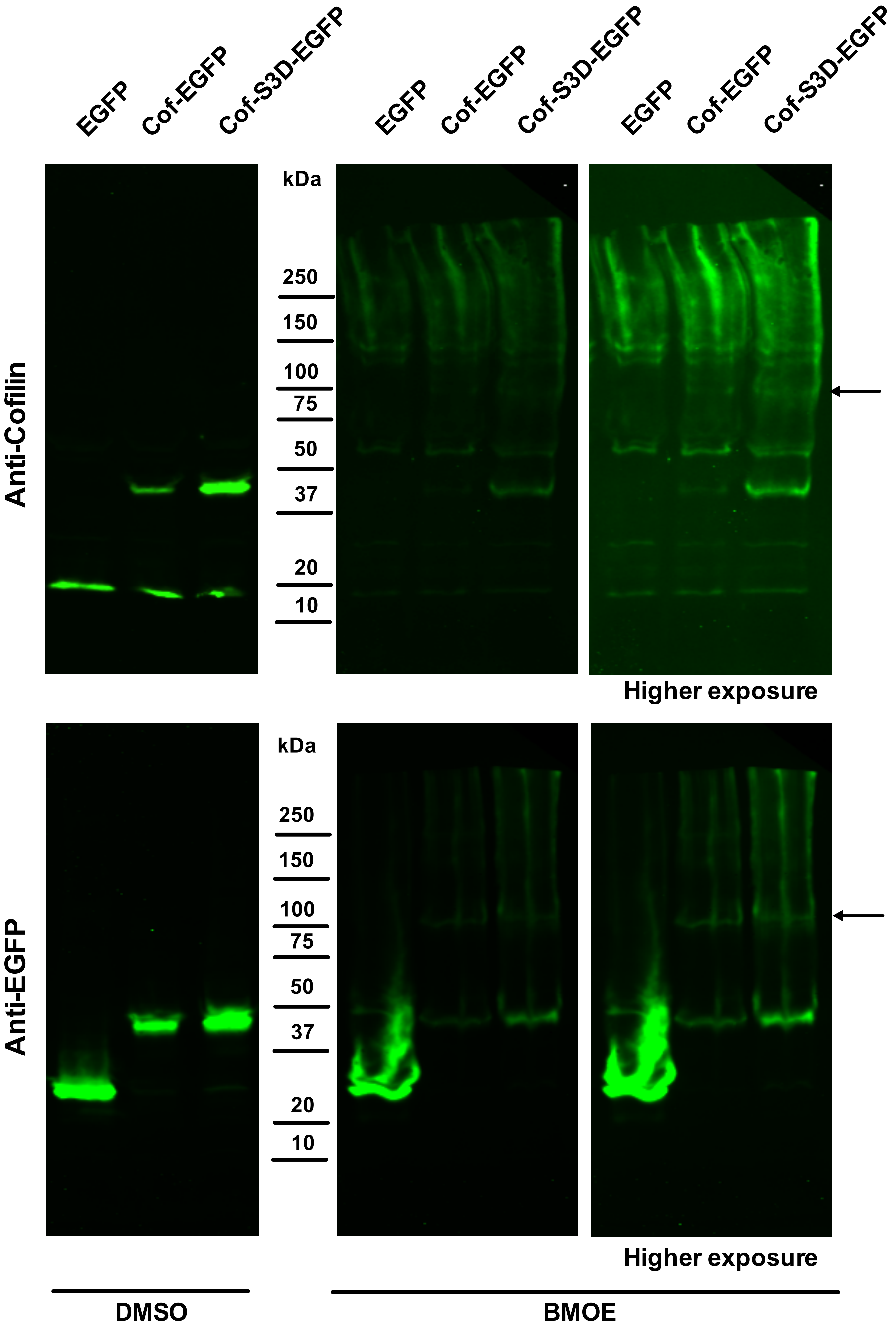
EGFP
Cof-EGFP
Cof-S3D-EGFP
EGFP
Cof-EGFP
Cof-S3D-EGFP
EGFP
Cof-EGFP
Cof-S3D-EGFP
Anti-Cofilin
kDa
250
150
100
75
50
37
20
10
Higher exposure
Anti-EGFP
kDa
250
150
100
75
50
37
20
10
Higher exposure
DMSO
BMOE
